# Down-Regulation of Transglutaminase 2 Stimulates Redifferentiation of Dedifferentiated Chondrocytes through Enhancing Glucose Metabolism

**DOI:** 10.3390/ijms18112359

**Published:** 2017-11-07

**Authors:** Kyoung-Won Ko, Bogyu Choi, Sunghyun Park, Yoshie Arai, Won Chul Choi, Joong-Myung Lee, Hojae Bae, In-Bo Han, Soo-Hong Lee

**Affiliations:** 1 Department of Biomedical Science, College of Life Science, CHA University, Seongnam-si 13488, Korea; glintk03@gmail.com (K.-W.K.); bgchoi725@gmail.com (B.C.); nightsky9836@naver.com (S.P.); ruddov@gmail.com (Y.A.); 2 Department of Orthopedic Surgery, Bundang Medical Center, CHA University, Seongnam-si 13496, Korea; wcosdoc@gmail.com (W.C.C.); drjmlee@naver.com (J.-M.L.); 3 College of Animal Bioscience and Technology, Department of Bioindustrial Technologies, Konkuk University, Seoul 05029, Korea; hojaebae@konkuk.ac.kr; 4 Department of Neurosurgery, CHA University, CHA Bundang Medical Center, Seongnam-si 13496, Korea

**Keywords:** chondrocytes, redifferentiation, transglutaminase 2, glycolytic metabolism

## Abstract

Expansion of chondrocytes for repair of articular cartilage can lead to dedifferentiation, making it difficult to obtain a sufficient quantity of chondrocytes. Although previous studies have suggested that culture in a three-dimensional environment induces redifferentiation of dedifferentiated chondrocytes, its underlying mechanisms are still poorly understood in terms of metabolism compared with a two-dimensional environment. In this study, we demonstrate that attenuation of transglutaminase 2 (TG2), a multifunctional enzyme, stimulates redifferentiation of dedifferentiated chondrocytes. Fibroblast-like morphological changes increased as TG2 expression increased in passage-dependent manner. When dedifferentiated chondrocytes were cultured in a pellet culture system, TG2 expression was reduced and glycolytic enzyme expression up-regulated. Previous studies demonstrated that TG2 influences energy metabolism, and impaired glycolytic metabolism causes chondrocyte dedifferentiation. Interestingly, TG2 knockdown improved chondrogenic gene expression, glycolytic enzyme expression, and lactate production in a monolayer culture system. Taken together, down-regulation of TG2 is involved in redifferentiaton of dedifferentiated chondrocytes through enhancing glucose metabolism.

## 1. Introduction

Articular cartilage is a unique avascular and aneural tissue in which chondrocytes are embedded in the extracellular matrix (ECM), which is mainly comprised of collagen, glycosaminoglycans (GAGs), and other glycoproteins. Articular chondrocytes maintain articular cartilage homeostasis by remodeling ECM molecules, including type II collagen and proteoglycans. Molecular composition of the ECM also influences the specific functions of articular chondrocytes [1,2,3]. Chondrocytes can exist under low oxygen conditions due to the avascular environment. Thus, glucose metabolism is the main energy source of chondrocytes [4,5,6,7]. Previous studies reported that suppression of glucose metabolism using an inhibitor of glycolytic enzymes can induce dedifferentiation of chondrocytes [8,9].

Since articular cartilage has low self-healing capacity, treatment of cartilage lesions after injury is important to restore cartilage-specific function. Therefore, different therapeutic strategies have been suggested for the treatment of impaired cartilage. Among them, autologous chondrocyte implantation (ACI) is a well-established therapy for patients with knee cartilage damage [10,11]. However, long-term expansion and serial passaging, which are required to obtain a sufficient amount of cells for therapy, lead to dedifferentiation of chondrocytes [12,13]. Dedifferentiation of chondrocytes gives rise to gradual loss of chondrogenic markers and chondrogenic ECM molecules such as type II collagen and GAG. Therefore, many studies have tried to restore the chondrogenic potential of dedifferentiated chondrocytes after serial passages by supplementing growth medium with defined factors [14,15,16,17,18,19], regulating cell density [20,21,22,23], cultivating cells in a more cartilage-like environment [24,25,26]. Three-dimensional (3D) culture systems such as pellet culture have been widely used to induce redifferentiation of dedifferentiated chondrocytes [27,28,29]. In terms of glucose metabolism, however, the molecular mechanism of redifferentiation in a 3D culture system is largely unknown.

Transglutaminase 2 (TG2), a stress-inducible factor, is a ubiquitously expressed multifunctional enzyme that catalyzes the post-translational modification of proteins in a Ca^2+^-dependent manner [30,31]. In addition to its various enzymatic activities, TG2 is associated with many cellular processes such as cell adhesion [32,33,34], survival, proliferation [35], migration [36,37] and autophagy [38,39]. Recently, it has been reported that TG2 modulates the transcription of critical genes including PPARγ coactivator-1alpha (PGC-1alpha) and cytochrome C that are important for function and biogenesis of mitochondria and regulates cellular energy metabolism [40,41]. Interestingly, increased TG2 expression is related with physiologic maturation to hypertrophy of growth plate chondrocytes. Chondrocyte hypertrophic differentiation is the gradual development process from chondrogenic differentiation to cartilage mineralization, but chondrocyte hypertrophy-like changes play a role in early and late stage osteoarthritis (OA) [42]. Moreover, it was reported that TG2 expression is related to articular chondrocyte hypertrophy and is a biomarker of OA severity [43].

In the present study, we observed gradual up-regulation of TG2 expression in chondrocytes during serial passage. However, elevation of TG2 expression in dedifferentiated chondrocytes was significantly diminished in a pellet culture system. Based on this link between TG2 and redifferentiation of dedifferentiated chondrocytes, we focused on alteration of energy metabolism by TG2 in a pellet culture system.

## 2. Results

### 2.1. TG2 Expression is Associated With Dedifferentiation of Chondrocytes

**Figure 1:**
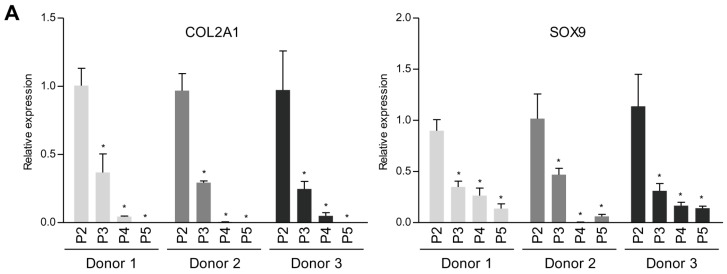

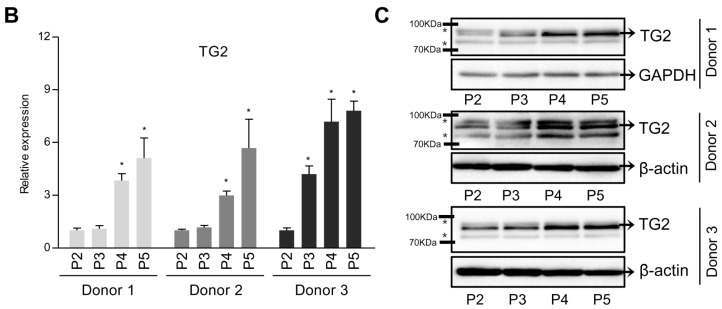
Induction of TG2 in dedifferentiated chondrocytes. Human articular chondrocytes were passed until passage 5 in normal growth medium. (**A**) COL2A1, SOX9 and (**B**) TG2 mRNA expression levels in human articular chondrocytes were measured by real-time qPCR. * *p* < 0.05. *n* = 3. (**C**) TG2 protein expression levels in human articular chondrocytes were detected by Western blotting using anti-TG2 polyclonal antibody. Asterisks denote other TGs. TG2 antibody cross-reacts with other transglutaminases. GAPDH and β-actin expressions were examined as a loading control.

To determine the TG2 expression level during dedifferentiation of chondrocytes, we first analyzed TG2 expression levels after serial passages in monolayer. Interestingly, TG2 expression in human articular chondrocytes from 3 different donors increased in a passage-dependent manner (Figure 1).

**Figure 2:**
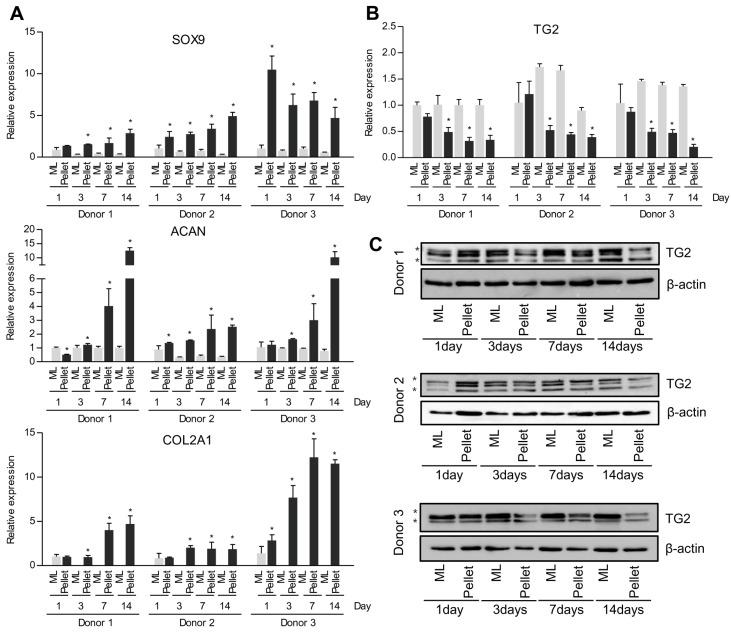
Changes in chondrogenic marker and TG2 expression in pellet culture system. Human articular chondrocytes were passaged up to 4 in monolayer culture. Cells were detached and cultivated in monolayer or pellet culture system for 14 days. (**A**) SOX9, COL2A1, ACAN and (**B**) TG2 mRNA expression levels were measured by real-time qPCR. Data represent mean values ± SD from at least three independent experiments. ML, monolayer. Pellet, pellet culture system. * *p* < 0.05. *n* = 3. (**C**) TG2 protein expression levels were examined by western blotting. Asterisks denote other TGs. β-actin was used as a loading control.

Redifferentiation of dedifferentiated chondrocytes was induced using pellet culture in a 3D environment. Subsequently, SRY (sex determining region Y)-box 9 (SOX9), collagen type II (COL2A1) and aggrecan (ACAN) mRNA expression levels were compared to their counterparts in monolayer culture. As shown in Figure 2A, expression of chondrogenic markers (SOX9, COL2A1 and ACAN) was significantly higher in the pellet culture system (Figure 2A) compared to monolayer culture. Next, TG2 expression in the 3D pellet culture system significantly decreased at both mRNA and protein levels (Figure 2B,C). These results indicate that TG2 expression is highly related to dedifferentiation of chondrocytes after serial passage.

### 2.2. Alteration of Energy Metabolism in Pellet Culture System

**Figure 3:**
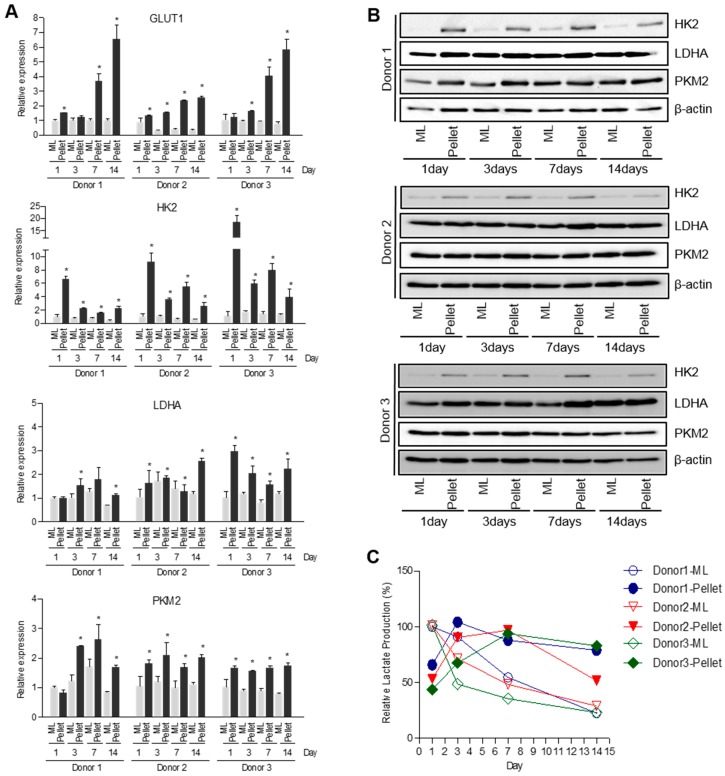
Alteration of energy metabolism in pellet culture system. Passage 5 human articular chondrocytes were cultured in monolayer or pellet culture system for 14 days. (**A**) GLUT1, HK2, LDHA and PKM2 mRNA expression levels were quantified by real-time qPCR. Data represent mean values ± SD from at least three independent experiments. * *p* < 0.05. *n* = 3. (**B**) HK2, LDHA and PKM2 protein expression levels were Western blot analysis. β-actin was used as a loading control. (**C**) Extracellular lactate levels secreted by cells in monolayer or pellet culture systems. Lactate concentration of monolayer culture for 1 day samples was assigned as 100%. ML, monolayer. Pellet, pellet culture system.

Articular chondrocytes are adapted to low oxygen environments in vivo and generate energy through glycolysis rather than oxidative phosphorylation in mitochondria [6]. We next compared the expression levels of genes involved in the glycolytic pathway between 2D monolayer and 3D pellet culture systems. Expression of glycolysis-related genes such as glucose transporter 1 (GLUT1), hexokinase 2 (HK2), lactate dehydrogenase A (LDHA) and pyruvate kinase muscle isozyme M2 (PKM2) largely increased in pellet culture compared to monolayer culture (Figure 3A,B). In addition, production of lactate, the end product of glycolysis, was much higher in monolayer culture on day 1 of the initial stage, after which it drastically decreased. After 3 days, 3D pellet culture induced higher production of lactate than 2D monolayer culture (Figure 3C). Taken together, these results suggest that metabolic change undergoes during redifferentiation of dedifferentiated chondrocytes in a pellet culture system.

### 2.3. Down-Regulation of TG2 Enhances Glycolytic Pathway in Chondrocytes

**Figure 4:**
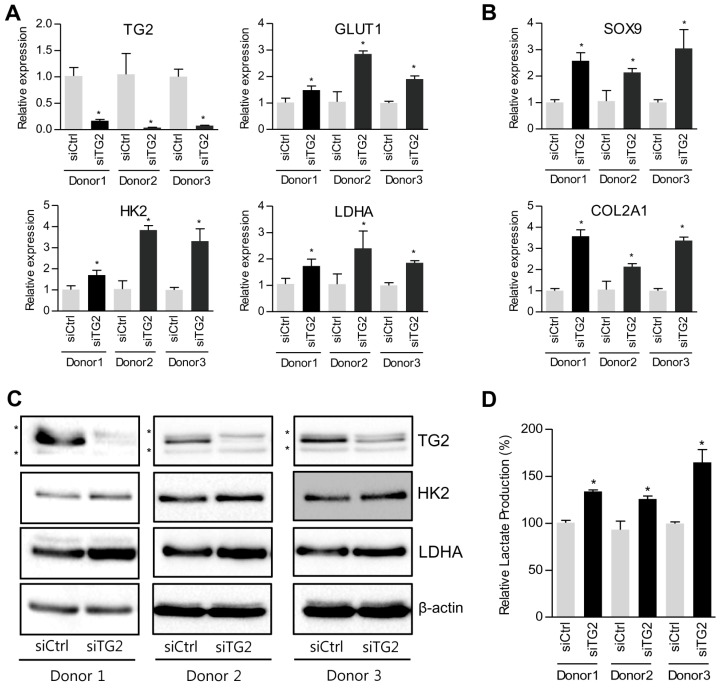
Metabolic reprogramming by TG2 knockdown in dedifferentiated chondrocytes. Passage 5 human articular chondrocytes were transfected with TG2-specific siRNA or non-silencing control siRNA. (**A**) TG2, GLUT1, HK2 and LDHA mRNA levels were quantified by real-time qPCR. (**B**) The mRNA expression levels of chondrogenic markers (SOX9 and COL2A1) were measured by real-time qPCR. (**C**) The protein expression levels TG2, HK2 and LDHA were quantified by western blot analysis. Asterisks denote other TGs. β-actin was used as a loading control. (**D**) Extracellular lactate levels secreted by cells transfected with control siRNA or TG2 siRNA. Lactate concentration of non-silencing control siRNA transfected cell samples was assigned as 100%. Data represent mean values±SD from at least three independent experiments. * *p* < 0.05. *n* = 3.

Since TG2 modulates energy metabolism in various cell types [41,44], we hypothesized that TG2 ablation in a pellet culture system can stimulate energy metabolism by regulating expression of glycolysis-related genes. siRNA-mediated knockdown of TG2 expression in dedifferentiated chondrocytes, as confirmed by reduced expression of TG2, induced significant up-regulation of glucose metabolism-associated genes (GLUT1, HK2 and LDHA) as well as chondrocgenic genes (SOX9 and COL2A1) (Figure 4). Furthermore, down-regulation of TG2 in dedifferentiated chondrocytes significantly enhanced production of lactate, demonstrating that down-regulation of TG2 is able to promote redifferentiation of dedifferentiated chondrocytes through improving glucose metabolism.

## 3. Discussion

It is well known that expansion of isolated chondrocytes using conventional monolayer culture method leads to loss of chondrogenic potential, especially dedifferentiation [12,13]. A number of studies have suggested techniques to maintain chondrogenic potential during expansion or induce redifferentiation of dedifferentiated chondrocytes. Among them, three-dimensional (3D) cultures are widely used for redifferentiation of dedifferentiated chondrocytes since they are very simple and low-cost. In terms of glucose metabolism, however, the underlying molecular mechanism of 3D pellet culture remains unclear.

In this study, elevation of TG2 expression was found to be highly associated with dedifferentiation of chondrocytes by serial passage while down-regulation of TG2 in a 3D pellet culture system improved redifferentiation of dedifferentiated chondrocytes through enhancing glycolytic metabolism.

As shown in Figure 1, TG2 expression was drastically augmented in a passage-dependent manner in human articular chondrocytes from 3 donors. It has been reported that TG2 is predominantly localized to the cytosol, although it has been found in mitochondria, the nucleus, cell membrane, and extracellular matrix [45,46]. TG2 is able to modify mitochondrial function, signal transduction targets, and cell surface receptors, which regulate intracellular signaling molecules and extracellular matrix synthesis [47,48]. A previous study demonstrated the PDI function of TG2 in vivo based on analysis of TGM2 knock-out mice, which exhibit abnormalities in the mitochondrial respiratory chain and ATP production [49]. However, it is unclear how TG2 regulates mitochondria-dependent processes.

Since cartilage is an avascular tissue, oxygen deficiency confers a hypoxic environment in vivo (1–5% oxygen tension) [1]. Therefore, articular chondrocytes are well adapted to low oxygen tension, resulting in low mitochondrial mass and low oxygen requirements followed by high glycolytic rates for survival. Previously, monolayer-cultured chondrocytes were shown to have markedly increased mitochondria content compared to freshly isolated chondrocytes. Moreover, chondrocytes undergo drastic alteration of metabolic status from characteristically glycolytic to oxidative energy metabolism during monolayer expansion [50]. Recent studies indicate that TG2’s activity not only can modulate the assembly of respiratory chain complexes in mitochondria but are able to also modulate the transcription of critical genes including PGC-1alpha and cytochrome C that are important for function and biogenesis of mitochondria [40,41]. Elevation of oxidative capacity results from excessive production of reactive oxygen species (ROS), and imbalance between ROS generation and antioxidant processes has been determined to be a cause of oxidative stress-mediated dedifferentiation and senescence of chondrocytes during monolayer expansion [51,52]. Further works need to identify how TG2 is involved in mitochondrial biosynthesis and oxidative stress-induced dedifferentiation and senescence in chondrocytes during monolayer culture.

It has been reported that TG2 expression is mainly controlled at the transcription level [53], and the TG2 promoter has response elements of TNF-α [54,55], IL-1β [56], and IL-6 [57]. Pro-inflammatory cytokines such as TNF-α and IL-1β inhibit expression of master chondrogenic factor SOX9, resulting in down-regulation of chondrocyte-specific markers such as collagen type II [58]. Recently, Johnson et al. reported that TG2 is an essential mediator of chondrocyte hypertrophy and calcification in response to both ATRA and IL-1β using TG2 knockout mice [56]. However, further studies are required to determine the upstream regulator of TG2 expression in dedifferentiated chondrocytes after serial passage.

Elevated TG2 expression of dedifferentiated chondrocytes began to decrease in the 3D pellet culture system (Figure 2). In contrast, expression of glucose metabolism-related genes (GLUT1, HK2, LDHA and PKM2) and lactate production increased in the 3D pellet culture system (Figure 3). GLUT1 is the transmembrane glucose transporter protein that mediate glucose uptake. Since HK2, LDHA and PKM2 are important enzymes that directly modulate glycolysis [59,60], we identified expression of HK2, LDHA and PKM2 in both mRNA and protein levels. Generally, the 3D pellet culture is widely used since it is very simple and favorable to maintain chondrogenic phenotype and improve redifferentiation of dedifferentiated chondrocytes. However, it is not yet clear which factors are involved in the redifferentiation process in a 3D environment. Interestingly, Hunt et al. reported reduction of mitochondrial activity in a 3D culture system [61]. Since TG2 is directly or indirectly implicated in mitochondrial homeostasis [62], cells lacking TG2 alter cellular energy metabolism towards aerobic glycolysis in an attempt to survive [41]. Accordingly, we hypothesized that down-regulation of TG2 expression in the 3D pellet culture system could lead to alteration of cellular energy metabolism. As shown in Figure 4, we observed that down-regulation of TG2 expression in dedifferentiated chondrocytes was responsible for increased expression of metabolic enzymes (HK2, PKM2, LDHA and GLUT1), resulting in enhancement of lactate production. Moreover, chondrogenic markers SOX9 and COL2A1 were restored in dedifferentiated chondrocytes by down-regulation of TG2. These results imply that TG2 reduction in a 3D environment improves redifferentiation of dedifferentiated chondrocytes through energy metabolism rearrangement. Although we did not clarify whether or not TG2 can directly modulate cellular metabolism and induce chondrogenic factors, increased chondrogenic potential can be attributed to metabolism-associated TG2 expression, which should be clarified in the future.

## 4. Materials and Methods

### 4.1. Ethics Statement

The study was approved by the institutional review board and ethics committee of CHA Bundang Medical Center (BD2014-07-097). A written informed consent about this experiment was obtained from all subjects.

### 4.2. Chondrocyte Isolation

Chondrocytes from 3 donors (Donor 1; 58-year-old female, Donor 2; 61-year-old male and Donor 3; 72-year-old male) were isolated from the undamaged region of osteoarthritic (OA) cartilage from total knee arthroplasty. Cartilage was separated from the subchondral bone and cut into 2 × 2-mm pieces using a surgical blade. Cartilage pieces were washed three times with HBSS (Hyclone, Logan, UT, USA) containing 2% antibiotic/antimycotic (Hyclone) and then digested at 37 °C, 5% CO_2_ in DMEM (Hyclone) containing 0.25 mg/mL of collagenase (Sigma, St. Louis, MO, USA). After 15 h, undigested tissue was removed from cells using a 45 μm cell strainer and plated in culture plates. Isolated cells were cultured in humidified air with 5% CO_2_ at 37 °C. Culture medium consisted of DMEM/Low glucose (Hyclone), 10% fetal bovine serum (FBS, Hyclone), and 100 units/mL of penicillin-streptomycin (Hyclone).

### 4.3. Pellet Preparation and Cultivation

For pellet culture, 200,000 cells (passage 4) per pellet were centrifuged at 500 rpm for 5 min in conical tubes in chondrogenic differentiation medium. The tubes were placed in a humidified atmosphere at 37 °C and 5% CO_2_ with the lid loosely closed. Chondrogenic differentiation medium consisted of high glucose medium, 1% (*v*/*v)* insulin transferrin selenium A (Gibco, Grand island, NY, USA), 50 μg/mL of ascorbic acid (Sigma), 100 nM dexamethasone (Sigma), and 10 ng/mL of TGF-β1 (Millipore, Schwalbach, Germany).

### 4.4. Western Blot Analysis

Cells or pellets were washed twice with PBS (Hyclone) and lysed in RIPA buffer (Cell Signaling Technology, Danvers, MA, USA). Protein concentration was determined using bicinchoninic acid (BCA) protein assay (Thermo Scientific, Rockford, IL, USA). Total protein extracts were separated by SDS-PAGE and electroblotted onto a nitrocellulose membrane. After blocking in 5% non-fat dry milk in Tris-buffered Saline Tween 20 (Biosesang, Gyeonggi-do, Korea), blots were incubated with rabbit polyclonal anti-TG2 antibody (Abcam, Cambridge, UK), rabbit polyclonal anti-Hexokinase 2 antibody, rabbit polyclonal anti-PKM2 antibody, and rabbit polyclonal anti-LDHA antibody (Cell Signaling Technology). After washing, secondary antibody (HRP-conjugated anti-rabbit immunoglobulin) was added and visualized using an ECL prime (GE Healthcare, Little Chalfont, UK).

### 4.5. RNA Isolation and Real Time Quantitative PCR

Total RNA was isolated using Trizol (Ambion, TX, USA) according to the manufacturer’s instructions, after which cDNA was synthesized with a PrimeScript RT reagent kit (Takara, Tokyo, Japan). Real-time quantitative PCR was performed using Power SYBR^®^ Green PCR Master Mix (Applied Biosystems, San Francisco, CA, USA). ABI StepOnePlus Real-Time PCR System was used for amplification using the following cycle conditions: 95 °C for 10 min × 1 cycle and 95 °C for 15 s, followed by 60 °C for 30 s × 40 cycles. Primer pairs for *TG2*, *ACAN*, *COL2A1*, *SOX9*, *GLUT1*, *HK2*, *PKM2*, *LDHA* and *18s rRNA* are shown in Table 1. Data were analyzed using the ΔΔ*C*_t_ method, mRNA expression was normalized to 18S rRNA as a housekeeping gene, and gene expression was calculated as a fold change.

### 4.6. Lactate Assay

For assessment of lactate production, medium was collected and diluted 1:100 in lactate assay buffer. The amount of lactate present in the media was measured using a Lactate Assay Kit (Biovision, Mountain View, CA, USA) according to the manufacturer’s instructions. The concentrations of lactate were normalized to protein concentration.

### 4.7. Knockdown Experiment

For the siRNA transfection experiments, cells were seeded in a 12-well plate. After 24 h, TG2 siRNA (Bioneer, Daejeon, Korea) was transfected into cells using Lipofectamine RNA iMAX reagent (Invitrogen, Carlsbad, CA, USA). Transfected cells were incubated for 72 h and subjected to various analyses.

### 4.8. Statistical Analysis

All data were expressed as the mean ± S.D. Student’s *t*-test was performed to compare two groups. One-way ANOVA with post-hoc tukey tests was used to analyze multiple groups. The *p* values less than 0.05 were considered as significant.

## 5. Conclusions

It is concluded that TG2 is a negative regulator of chondrogenic potential in chondrocytes during expansion. The 3D environment is able to inhibit elevation of TG2 expression of dedifferentiated chondrocytes. On the other hand, regulation of TG2 and/or TG2-dependent signal transduction during monolayer expansion will provide a useful tool to reduce dedifferentiation of chondrocytes.

## Figures and Tables

**Table 1:** Nucleotide sequences of primer pairs for real-time PCR.

Gene	Human Primer Sequence	
*TG2*	Sense	5′-GGCACCAAGTACCTGCTCA-3′
	Antisense	5′-AGAGGATGCAAAGAGGAACG-3′
*ACAN*	Sense	5′-GCCTGCGCTCCAATGACT-3′
	Antisense	5′-ATGGAACACGATGCCTTTCAC-3′
*COL2A1*	Sense	5′-CACGTACACTGCCCTGAAGGA-3′
	Antisense	5′-CGATAACAGTCTTGCCCCACTT-3′
*SOX9*	Sense	5′-CCCCAACAGATCGCCTACAG-3′
	Antisense	5′-GAGTTCTGGTCGGTGTAGTC-3′
*GLUT1*	Sense	5′-GGTTGTGCCATACTCATGACC-3′
	Antisense	5′-CAGATAGGACATCCAGGGTAGC-3′
*HK2*	Sense	5′-TCCCCTGCCACCAGACTA-3′
	Antisense	5′-TGGACTTGAATCCCTTGGTC-3′
*PKM2*	Sense	5′-CGTCTGAACTTCTCTCATGGAA-3′
	Antisense	5′-ATGGGGTCAGAAGCAAAGC-3′
*LDHA*	Sense	5′-GCAGATTTGGCAGAGAGTATAATG-3′
	Antisense	5′-GACATCATCCTTTATTCCGTAAAGA-3′
*18S rRNA*	Sense	5′-CTTCCACAGGAGGCCTACAC-3′
	Antisense	5′-CGCAAAATATGCTGGAACTTT-3′

## Abbreviations

3D culture system	Three-Dimensional Culture System
ACI	Autologous Chondrocyte Implantation
ECM	Extracellular Matrix
GAG	Glycosaminoglycan
TG2	Transglutaminase 2
SOX9	SRY (Sex Determining Region Y)-Box 9 (SOX9)
COL2A1	Collagen Type II
ACAN	Aggrecan
GLUT1	Glucose Transporter 1
HK2	Hexokinase 2
LDHA	Lactate Dehydrogenase A
PKM2	Pyruvate Kinase Muscle isozyme M2
TGF-β1	Transforming Growth Factor Beta 1
